# Editorial Note: Development of New Mouse Lung Tumor Models Expressing EGFR T790M Mutants Associated with Clinical Resistance to Kinase Inhibitors

**DOI:** 10.1371/journal.pone.0323763

**Published:** 2025-05-06

**Authors:** The PLOS One Editors

After publication of this article [1], concerns were raised regarding results presented in Fig 5B. Specifically, the Ki-67 C/L858R+T790M 0D and the Ki-67 C/L858R+T790M 8D panels appear similar.

The corresponding author stated that an error occurred in the preparation of Fig 5B, but that the original image data underlying the results are no longer available. In the absence of these data, the concerns for Fig 5B cannot be fully resolved, and the Fig 5B results should be interpreted with caution.

The *PLOS One* Editors issue this Editorial Note to inform readers of the unresolved issue with Fig 5B.
